# Prevalence and correlates of disability in older adults, Ghana: evidence from the Ghana 2021 Population and Housing Census

**DOI:** 10.1186/s12877-023-04587-6

**Published:** 2024-01-11

**Authors:** Martin Wiredu Agyekum, Grace Frempong Afrifa-Anane, Frank Kyei-Arthur

**Affiliations:** 1 Institute for Educational Research and Innovation Studies, University of Education Winneba, Winneba, Ghana; 2 Department of Environment and Public Health, University of Environment and Sustainable Development, Somanya, Ghana

**Keywords:** Disability, Older adults, Prevalence, Ghana, Ghana 2021 Population and Housing Census

## Abstract

**Background:**

Although there are studies on disabilities in older persons, most of these studies have been carried out in developed countries. Hence, there are limited studies on disability in older adults in sub-Saharan Africa, including Ghana. The few studies that have examined the prevalence and correlates of disability in older adults used survey data for their analyses. To contribute to addressing this knowledge gap that has arisen, this study used a national census, the 2021 Ghana Population and Housing Census, to examine the prevalence and correlates of disability in older adults in Ghana.

**Methods:**

The 2021 Ghana Population and Housing Census data was used for this study. A sample size of 197,057 Ghanaians aged 60 years and above was used for this study. The Washington Group questions on disability were used to measure disability by asking older adults about their difficulties in performing the six domains of disability (physical, sight, intellectual, hearing, self-care, and speech). A multinomial logistic regression housed in STATA was used to analyse the correlates of disability in older adults in Ghana. A p-value less than 0.05 was used for statistical significance.

**Results:**

The results show that slightly more than one-third (38.4%) of the older adults were disabled. In terms of the number of disabilities in older adults, 16.9% had one disability condition, while 2.4% had six disability conditions. Also, 9.4% had two disability conditions. Older adults who were females, aged 70–79 years and 80 years and above, resided in rural areas, with primary, JHS/Middle, SHS, unaffiliated with religion, ever married and never married, unemployed, and belonged to the middle and rich households were more likely to have a disability condition. Also, older adults residing in the Middle and Northern zones, having no health insurance, and using clean cooking fuel were less likely to have a disability condition.

**Conclusions:**

The results show that socio-demographic and household factors were associated with disability in older adults in Ghana. Hence, policymakers and researchers should target these factors when designing appropriate policies, programmes, and interventions to improve the wellbeing of older adults.

**Supplementary Information:**

The online version contains supplementary material available at 10.1186/s12877-023-04587-6.

## Background

The proportion of older adults (60 years and above) continues to grow worldwide due to an increase in longevity and a decline in fertility [1–3]. In Ghana, the proportion of older adults has increased by 2% from 4.5% (213,477 older adults) in 1960 to 6.5% (1,991,736 older adults) in 2021 [4]. According to projections, the population of older adults in Ghana is expected to increase to 2.8 million by the year 2030 and further rise to 6.3 million [5]. While population ageing is a phenomenon that should be acknowledged and appreciated, studies have linked population ageing with increased risk of disabilities, chronic diseases, fall and fall-related injuries, and mental health issues [6–9].

Disability refers to restrictions in performing basic activities of daily life [10]. It is multi-dimensional and can be measured by several indicators including impairments, participation restrictions, and functional difficulties [11]. The Washington Group measures disability using levels of difficulties in six domains namely: seeing, hearing, physical (walking or climbing stairs), intellectual (remembering or concentrating), self-care and speech (communicating) [11].

Disability is a public health issue since it can negatively impact the health of older adults, restrict their participation in activities, and increase their dependence on others [12–14]. Globally, studies have found associations between socio-demographic factors (e.g., age, sex, place of residence, marital status, and wealth status), health factors (self-reported health and chronic conditions) and disability in older adults. For example, with regard to age, studies have linked increasing age with a higher risk of disability [2, 6, 15, 16] since the process of ageing is associated with functional decline and sensory loss [17]. Biritwum et al.’s [18] study among older adults in six countries (Ghana, South Africa, China, India, Russia and Mexico) found that the prevalence of disability increased with age. Similarly, Darkwah et al.’s [19] study among Ghanaian older adults found a positive association between age and disability.

Sex has also been identified as a correlate of disability in older adults. Studies have found that female older adults disproportionately bear a higher prevalence of disability than male older adults [16, 18, 19]. Malik’s [20] study among Indian older adults found that female older adults were more likely to be functionally disabled than male older adults. Also, a systematic review by Mangipudi et al. [21] found that female older adults had a higher risk of disability than male older adults. The higher prevalence of disability in female older women could be attributed to social and economic inequalities [22].

Regarding place of residence, the findings are mixed. In some studies, the prevalence of disability was higher in older adults residing in rural areas [15, 23], while it was higher in older adults residing in urban areas in other studies [24, 25].

Studies have documented a lower prevalence of disability in married older adults than those who have never and ever married [15, 23]. Wang et al.’s [26] study among older adults in China found that married older adults recorded better functional disability outcomes than never and ever married older adults. In South Africa, a study by Xavier Gómez-Olivé et al. [27] found that never married older adults had a higher likelihood of having disability than those married. In addition, studies have found an inverse association between wealth status and disability [28–30]. Biritwum et al.’s [18] study found that the prevalence of disability decreased as wealth status increased.

Poor/ill health is associated with higher prevalence of disability. Wandera et al.’s [15] study in Uganda found that older adults with ill health reported higher disability than those with good health. Similarly, a study by Debpuur et al.’s [31] in Ghana among older adults in Kassena-Nankana district found that poor health was associated with higher prevalence of disability.

Although there are studies on disabilities in older persons, most of these studies have been carried out in developed countries [26, 29, 30]. Thus, there are limited studies on disability in older adults in sub-Saharan Africa, including Ghana. Also, the few studies that have examined the prevalence and correlates of disability in older adults [18, 19, 31] used survey data for their analyses. Thus, there is lack of studies in Ghana using data that encompasses the entire country to examine disability and its correlates in older adults aged 60 years and above as well using the Washington Group questions on disability to measure disability. To contribute to addressing this knowledge gap that has arisen, this study examined the prevalence and correlates of disability in older adults in Ghana using the 2021 Ghana Population and Housing Census (2021 GPHC) which uses the Washington Group questions on disability to measure disability. The use of the 2021 GPHC data will enhance the understanding of disability in older adults in Ghana, and consequently, help policymakers and researchers to design appropriate policies, programmes, and interventions to improve the wellbeing of older adults.

## Methods

### Study setting

Ghana has a population of 30.8 million population as of 2021 [4]. It has 16 administrative regions and Greater Accra is the regional capital. Ghana is bounded by Burkina Faso in the North, South by the Gulf of Guinea, East by Togo and in the West by Cote d’Ivoire. More than half (56.7%) of the population reside in urban areas [4]. In addition, the majority of Ghana’s population (58.1%) are aged 15–59 years, 35.4% are aged 0–14 years, while 6.5% are aged 60 years and above [4].

### Study design and sampling procedure

The study utilised 10% of the 2021 GPHC data (3,083,572 persons) conducted by the Ghana Statistical Service, which is available to the general public, including researchers. The 10% of the 2021 GPHC data is nationally representative of the entire population, including older adults. The 2021 GPHC is a complete enumeration of all persons/households in Ghana, irrespective of their nationality to provide updated information on demographic, social, economic, housing and dwelling characteristics in the entire country. It is the sixth census conducted in Ghana after independence but the first to fully use Computer Assisted Personal Interview (CAPI) and Batch Program for Data Entry and Cleaning [4]. This study used data from Ghanaians aged 60 years and above (older adults) with a sample size of 197,057.

### Description and conceptualisation of variables

#### Dependent variable

The dependent variable for the study was disability. The Washington Group questions on disability was used to measure disability in older adults on six domains namely physical (walking or climbing stairs), sight (seeing), intellectual (remembering or concentrating), hearing, self-care, and speech (communicating) during the 2021 GPHC. During the census, older adults were asked about their difficulties in performing the above-mentioned domains. The responses were as follows: no difficulty, some difficulty, a lot of difficulties, and cannot do it at all. No difficulty was coded as “0” and any form of difficulty (some difficulty, a lot of difficulties, and cannot do it at all) was also coded as “1”. All these responses were put together to create a continuous variable which ranged from 0 to 6. The continuous variable was recategorised into 3 categories: “0”, “1” and “2 and more”. Zero (0) represent no disability, while one (1) represent a disability condition. Two or more (2 or more) represent 2 or more disability conditions.

#### Independent variables

The independent variables included age, sex, education, marital status, employment status, region of residence, place of residence, religious affiliation, household wealth, health insurance status, and cooking fuel. The age of the older adults was classified as 60–69 years, 70–79 years, and 80 years and above. Sex of respondents was categorised as males and females. The highest level of education was coded as no education, primary, Junior High School (JHS)/Middle, Senior High School (SHS) and Tertiary. Marital status was classified as currently married, never married and ever married. Ever married consist of older adults who are widowed, had separated or divorced. Employment status was measured by respondents who engaged in any economic activity seven (7) days before the census night for at least an hour. Respondents who responded yes were classified as employed, and those who responded no were classified as unemployed.

For the region of residence, the data was collected in all the sixteen regions (Greater Accra, Oti, Volta, Central, Western, Western North, Eastern, Ashanti, Bono East, Ahafo, Bono, Savanna, Northern, Upper East, Upper West and North East) in Ghana. This was re-categorised as Coastal (Greater Accra, Oti, Volta, Central, Western, Western North), Middle (Eastern, Ashanti, Bono East, Ahafo, Bono) and Northern (Savanna, Northern, Upper East, Upper West and North East) zones. Place of residence was categorised as rural and urban. Ghana Statistical Service defined a rural area as an area with less than 5,000 population. With regard to religious affiliation, respondents were asked the religion that they were affiliated to. Those who belonged to any religious affiliation were classified as affiliated (Christians, Moslems, Traditionalists and other religions), whiles those who do not belong to any religion was classified as not affiliated.

The household wealth was computed from ownership of household assets, including radio/stereo, television (digital and analog), telephone (fixed and cordless), bicycle, canoe, and outboard motor. Other household assets included fridge, deep freezer, desktop computer, laptop, tricycle, motor cycle, private car/truck, tractor, cart, donkey/camel/mule, and home theatre. Principal components analysis was used to generate factor scores for each household asset. A standardised score was used to divide the household wealth score into lowest, second, middle, fourth, and highest. The lowest and second were reclassified as poor, while the fourth and highest were reclassified as rich. The middle was left alone. Consequently, the household wealth had three categories: poor, middle and rich. More details about the computation of household wealth can be found on the ICF International website [32].

Health insurance status was categorised as yes for those with health insurance and no for those without health insurance. Cooking fuel was categorised as clean (liquified petroleum gas, biogas, electricity and cooking gel) and polluting (wood, kerosene, charcoal, crop residue, sawdust and animal waste). More details about the variables are found in Table S1 in the supplementary material.

### Data collection

The Ghana Statistical Service is mandated by law (Statistical Service Act, 2019: Act 1003) to conduct national censuses, and persons within the boundaries of Ghana are required by the same law to provide the needed information. The recruitment of enumerators for the 2021 GPHC was conducted via the Enumerator Bureau Recruitment Portal, an internet-based platform specifically designed to enlist individuals to serve as enumerators. Enumerators were required to indicate the districts in which they reside during the application process. The recruitment and training of enumerators took place within their respective districts, where they underwent a comprehensive 11-day training programme facilitated by experienced regional trainers. Seventy-five thousand and fifty individuals were recruited and underwent training nationwide.

The 2021 GPHC data were collected between 27th July and 8th August 2021. The data collection was conducted in languages that were understandable to the participants. The recruited enumerators were assigned to districts where they resided, making them more likely to be familiar with the participants’ languages. This familiarity greatly facilitated the process of data collection.

The entire country was demarcated into small geographic areas called enumeration areas (EAs) for data collection purposes, and enumerators collected the data from EAs. All households in Ghana were enumerated [4]. In each household, all usual household members and visitors who spent the census night (27th July, 2021) were enumerated.

### Data analysis

The characteristics of the respondents were described using descriptive statistics such as percentages and frequencies. Afterwards, a multinomial logistic regression housed in STATA was used to determine the factors associated with disability. A multinomial logistic regression was used because the dependent variable (disability) had three (3) categories (no disability, one disability, and two or more disabilities). Adjusted odds ratio (aOR) and 95% confidence intervals were used to describe and interpret the findings of the study. A p-value less than 0.05 was used for statistical significance.

## Results

### Description of the background characteristics of older adult

Table 1 shows the background characteristics of the older adults. More than half (55.6%) of them were aged 60–69 years, and the proportion of the older adults with a disability condition was higher for those older than 60–69 years. Those aged 80 and above constituted the highest proportion (44.9%) living with two or more disability condition compared to those of younger ages within the group under comparison (p ≤ 0.001).

**Table 1:** Description of background characteristics of older adults

			Disability conditions		
	Older adults	No disability	One disability	Two or more disabilities	
Variables	n = 197, 057 Frequency (%)	n = 121,451 Frequency (%)	n = 33,345 Frequency (%)	n = 42,261 Frequency (%)	p-value
**Age**					≤ 0.001
60–69	109,606 (55.6)	78,685 (71.8)	17,372 (15.9)	13,549 (12.3)	
70–79	54,027 (27.4)	30,044 (55.6)	10,284 (19.0)	13,699 (25.4)	
80 and above	33,424 (17.0)	12,722 (38.1)	5,689 (17.0)	15,013 (44.9)	
**Sex**					≤ 0.001
Male	85,226 (43.3)	56,681 (66.5)	13,820 (16.2)	14,725 (17.3)	
Female	111,831 (56.7)	64,770 (57.9)	19,525 (17.5)	27,536 (24.6)	
**Education**					≤ 0.001
No education	97,855 (49.6)	56,459 (57.7)	15,625 (16.0)	25,771 (26.3)	
Primary	13,970 (7.1)	8,031 (57.5)	2,770 (19.8)	3,169 (22.7)	
JHS/Middle	52,943 (26.9)	34,637 (65.4)	9,496 (18.0)	8,810 (16.6)	
SHS	21, 323 (10.8)	14,248 (66.8)	3,770 (17.7)	3,305 (15.5)	
Tertiary	10, 966 (5.6)	8,076 (73.7)	1,684 (15.3)	1,206 (11.0)	
**Marital status**					≤ 0.001
Currently married	105,587 (53.6)	72,097 (68.3)	16,666 (15.8)	16,824 (15.9)	
Ever married	86,904 (44.1)	46,560 (53.6)	15,944 (18.4)	24,400 (28.0)	
Never married	4,566 (2.3)	2,794 (61.2)	735 (16.1)	1,037 (22.7)	
**Employment status**					≤ 0.001
Employed	92,514 (47.0)	66,891 (72.3)	14,571 (15.7)	11,052 (12.0)	
Unemployed	104,543 (53.0)	54,560 (52.2)	18,774 (18.0)	31,209 (29.8)	
**Region of residence**					≤ 0.001
Coastal	86,198 (43.7)	52,000 (60.3)	15,100 (17.5)	19,098 (22.2)	
Middle	76,586 (38.9)	47,824 (62.5)	13,276 (17.3)	15,486 (20.2)	
Northern	34,273 (17.4)	21,627 (63.1)	4,969 (14.5)	7,677 (22.4)	
**Place of residence**					≤ 0.001
Urban	103,483 (52.5)	66,708 (64.5)	16,830 (16.3)	19,945 (19.2)	
Rural	93,574 (47.5)	54,743 (58.5)	16,515 (17.7)	22,316 (23.8)	
**Religious affiliation**					≤ 0.001
Affiliated	185,394 (94.1)	114,559 (61.8)	31,377 (16.9)	39,458 (21.3)	
Unaffiliated	11,663 (5.9)	6,892 (59.1)	1,968 (16.9)	2,803 (24.0)	
**Household wealth**					≤ 0.001
Poor	66,509 (33.7)	44,817 (67.4)	10,598 (15.9)	11,094 (16.7)	
Middle	50,011 (25.4)	29,996 (60.0)	9,044 (18.0)	10,971 (22.0)	
Rich	80,537 (40.9)	46,638 (57.9)	13,703 (17.0)	20,196 (25.1)	
**Health insurance status**					≤ 0.001
Yes	141,542 (71.8)	86,676 (61.2)	24,434 (17.3)	30,432 (21.5)	
No	55,515 (28.2)	34,775 (62.6)	8,911 (16.1)	11,829 (21.3)	
**Cooking fuel**					≤ 0.001
Polluting	139,612 (70.9)	83,657 (59.9)	24,185 (17.3)	31,770 (22.8)	
Clean	57,445 (29.1)	37,794 (65.8)	9,160 (16.0)	10,491 (18.2)	
**Total**	197, 057 (100.0)	121,451 (61.6)	33,345 (16.9)	42,261 (21.5)	

*Source*: 2021 GPHC

Also, there was more female older adults (56.7%) than males (43.3%), and significantly a higher proportion (24.6%) of the females had two or more disability conditions compared to their male counterparts (17.3%, p ≤ 0.001).

Regarding education, about half (49.6%) of older adults had no formal education, and a few (5.6%) had attained tertiary education. The highest proportion (57.7%) of older adults with no formal education had no disability, however, 26.3% had two or more disability condition. Among those with higher education, the majority (73.7%) had no disability but about a tenth had two or more disability conditions (p ≤ 0.001). More than half (53.6%) of the older adults were currently married, and most (28%) ever married older adults had two or more disability conditions than those currently married (15.9%, p ≤ 0.001).

Also, 53% of older adults were unemployed, and most (29.8%) of them had two or more disability conditions compared to those employed (12%, p ≤ 0.001). The highest proportion (43.7%) of the older adults resided in the Coastal zone, and a few lived in the Northern zone. Almost the same proportion of older adults in Coastal (22.2%) and Northern (22.4%) zones had two or more disability conditions (p ≤ 0.001).

More than half (52.5%) of the older adults resided in urban areas compared to those in rural areas (47.5%). A higher proportion (23.8%) of the rural residents had two or more disability conditions than those who resided in urban areas (19.2%, p ≤ 0.001). Also, a higher proportion of the older adults were religiously affiliated (94.1%) and had health insurance (71.8%). A little over one-fifth (21.3% and 21.5%, p ≤ 0.001) of older adults who were religiously affiliated and had health insurance had two or more disability conditions, respectively.

Two-fifths (40.9%) of older adults were from rich households, and most (42.1%) of those from rich households had one or more disability conditions than those from poor households (32.6%, p ≤ 0.001). In terms of cooking fuel, 7 out of 10 (70.9%) of the older adults’ households used polluting cooking fuel. A higher proportion of those who used polluting cooking fuel (40.1%) had one or more disability conditions than those whose households used clean cooking fuel (34.2%, p ≤ 0.001).

### Prevalence of disability in older adults

**Fig. 1:**
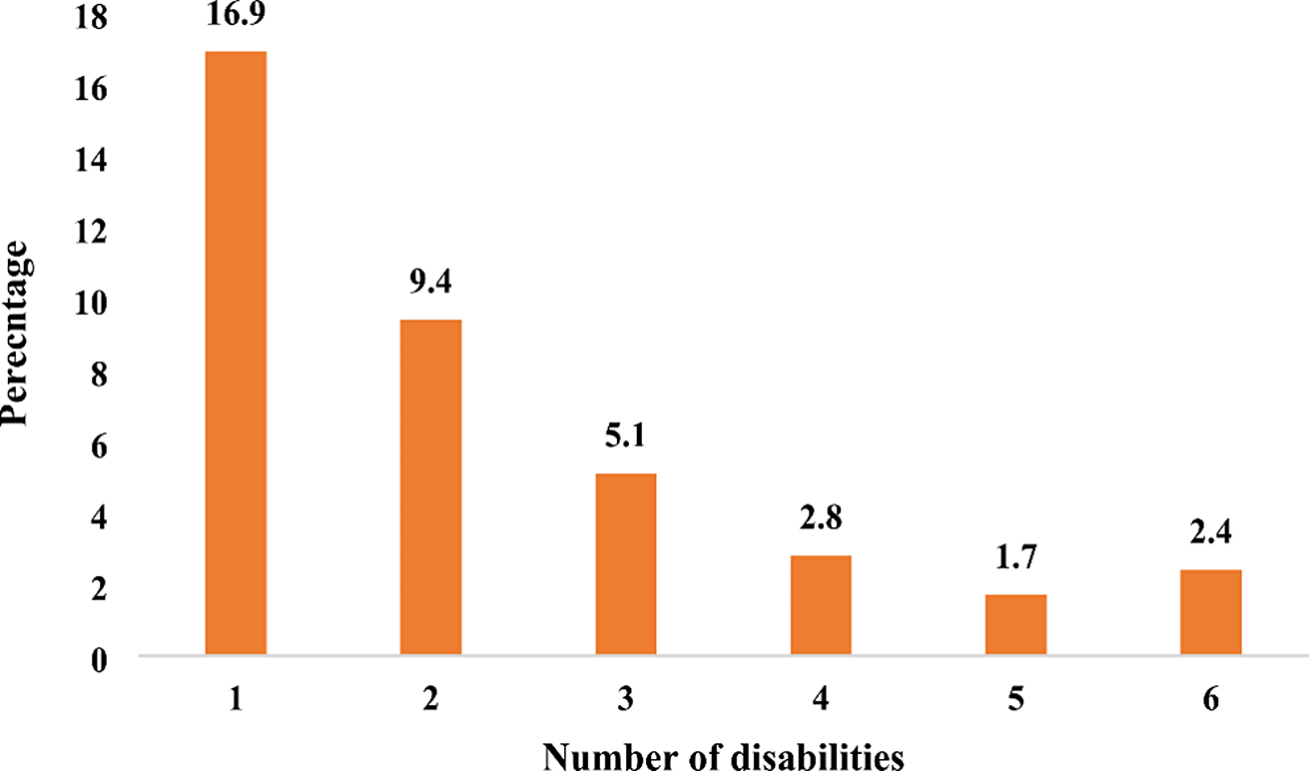
Number of disabilities in older adults. Sample size = 75,606. Source: 2021 GPHC

Generally, close to two-fifths (38.4%) of the older adults had a disability condition (Table 1). Figure 1 shows the number of disabilities in older adults. About 17.0% of older adults had one (1) disability condition and 2.4% had 6 disability conditions. The minimum and maximum number of disability condition is 0 and 6 respectively, with a mean of 0.9 and a standard deviation of 1.4.

### Factors associated with disability in older adults in Ghana

Table 2 illustrates the factors associated with disability in older adults in Ghana. The multinomial logistic regression shows that age, sex, education, marital status, employment status, region of residence, place of residence, religious affiliation, household wealth, health insurance, and cooking fuel were significantly associated with disability in older adults.

**Table 2:** Multinomial logistic regression of factors associated with disability in older adults in Ghana

	One disability vs. No disability		Two or more disabilities vs. No disability	
	aOR [95% CI]	p-value	aOR [95% CI]	p-value
**Age**				
60–69 (RC)				
70–79	1.43 [1.39–1.47]	≤ 0.001	2.08 [2.02–2.14]	≤ 0.001
80 and above	1.73 [1.67–1.80]	≤ 0.001	4.24 [4.10–4.38]	≤ 0.001
**Sex**				
Males (RC)				
Female	1.07 [1.03–1.10]	≤ 0.001	1.06 [1.03–1.09]	≤ 0.001
**Education**				
No education (RC)				
Primary	1.41 [1.34–1.48]	≤ 0.001	1.28 [1.22–1.34]	≤ 0.001
JHS/Middle	1.22 [1.18–1.26]	≤ 0.001	0.95 [0.91–0.98]	0.001
SHS	1.22 [1.17–1.28]	≤ 0.001	0.85 [0.81–0.89]	≤ 0.001
Tertiary	1.04 [0.97–1.10]	0.245	0.59 [0.55–0.63]	≤ 0.001
**Marital Status**				
Currently married (RC)				
Ever married	1.25 [1.21–1.28]	≤ 0.001	1.42 [1.38–1.46]	≤ 0.001
Never married	1.10 [1.02–1.20]	0.021	1.47 [1.36–1.59]	≤ 0.001
**Employment status**				
Employed (RC)				
Unemployed	1.49 [1.45–1.53]	≤ 0.001	2.63 [2.56–2.70]	≤ 0.001
**Region of residence**				
Coastal (RC)				
Middle	0.92 [0.89–0.94]	≤ 0.001	0.83 [0.81–0.86]	≤ 0.001
Northern	0.72 [0.69–0.75]	≤ 0.001	0.69 [0.67–0.72]	≤ 0.001
**Place of residence**				
Urban (RC)				
Rural	1.25 [1.22–1.28]	≤ 0.001	1.37 [1.33–1.40]	≤ 0.001
**Religious affiliation**				
Affiliated (RC)				
Unaffiliated	1.02 [0.97–1.08]	0.391	1.11 [1.05–1.17]	≤ 0.001
**Household wealth**				
Poor (RC)				
Middle	1.20 [1.16–1.25]	≤ 0.001	1.30 [1.25–1.34]	≤ 0.001
Rich	1.17 [1.13–1.21]	≤ 0.001	1.37 [1.33–1.42]	≤ 0.001
**Health insurance status**				
Yes (RC)				
No	0.91 [0.88–0.93]	≤ 0.001	0.96 [0.94–0.99]	0.009
**Cooking fuel**				
Polluting (RC)				
Clean	0.86 [0.83–0.89]	≤ 0.001	0.93 [0.90–0.96]	≤ 0.001

aOR = Adjusted odds ratio, CI = Confidence Interval, RC = Reference Category

*Source*: 2021 GPHC

The results show that the likelihood of having one disability, relative to having no disability, was 43% higher for older adults who were aged 70–79 year than for those aged 60–69 years. Similarly, those aged 70-79 years were 1.08% more likely to have two or more disability conditions than no disability, compared to those aged 60–69 years. Also, older adults who were 80 years and above had 73% higher likelihood of having one disability condition relative to no condition compared to those aged 60–69 years. Similarly, they were 324% more likely to have two or more disability conditions than no disability compared to those 60–69 years.

Female older adults were more likely to have one disability condition than no disability compared to males [aOR = 1.07; 95% CI = 1.03–1.10]. Likewise, females were 6% more likely [aOR = 1.06; 95% CI = 1.03–1.09] to have two or more disability conditions than no disability compared to males.

With regard to educational level, those who had primary, JHS/Middle, SHS and Tertiary education were more likely to have one disability condition than no disability compared to those who had no formal education. In contrast, those who had JHS/Middle, SHS and Tertiary education were less likely to have two or more disability conditions than no disability compared to those with no education.

For marital status, compared to older adults who were currently married, those who were ever married [aOR = 1.25; 95% CI = 1.21–1.28] and never married [aOR = 1.10; 95% CI = 1.02–1.20] had a higher likelihood of having a disability condition than no disability. On the other hand, compared to older adults who were currently married, older adults who were ever married [aOR = 1.42; 95% CI = 1.38–1.46] and never married [aOR = 1.47; 95% CI = 1.36–1.59] were more likely to have two or more disability conditions than no disability.

In addition, compared to older adults who were employed, those who were unemployed [aOR = 1.49; 95% CI = 1.45–1.53] were more likely to have one disability condition than no condition. Similarly, older adults who were unemployed were more likely to have two or more disability conditions than no condition, compared to those who were employed.

Regarding region of residence, compared to older adults who resided in the Coastal zone, older adults who resided in the Middle [aOR = 0.92; 95% CI = 0.89–0.94] and Northern [aOR = 0.72; 95% CI = 0.69–0.75] zones were 8% and 28% less likely to have one disability condition than no disability. Likewise, older adults who resided in the Middle [aOR = 0.83; 95% CI = 0.81–0.86] and Northern [aOR = 0.69; 95% CI = 0.67–0.72] zones were 17% and 31% less likely to have two or more disability conditions than no condition, compared to those who resided in the Coastal zone.

Also, compared to older adults living in the urban area, those living in the rural areas [aOR = 1.25; 95% CI = 1.22–1.28] were 25% more likely to have one disability condition than no condition. Similarly, older adults living in the rural areas [aOR = 1.37; 95% CI = 1.33–1.40] were 37% more likely to have two or more disability conditions than no disability compared to those living in the urban.

In terms of religious affiliation, older adults who were unaffiliated were 11% more likely [aOR = 1.11; CI 95%= 1.05–1.17] to have two or more disability conditions than no disability condition, compared to those who were affiliated.

Furthermore, compared to older adults who belonged to poor households, those who belonged to middle [aOR = 1.20; 95% CI = 1.16–1.25] and rich [aOR = 1.17; 95% CI = 1.13–1.21] households were 20% and 17% more likely to have one disability condition than no disability. Similarly, those who belonged to the middle and rich households were 30% and 37% more likely respectively to have two or more disability conditions than no disability, compared to their counterparts who belonged to poor households.

Older adults with no health insurance [aOR = 0.91; 95% CI = 0.88–0.93] were 9% less likely to have one disability condition than no disability, compared with those who had health insurance. Similarly, compared with those who had health insurance, older adults with no health insurance [aOR = 0.96; 95% CI = 0.94–0.99] were 4% less likely to have two or more disability conditions than no disability.

With cooking fuel, older adults whose households used clean cooking fuel [aOR = 0.86; 95% CI = 0.83–0.89] were 14% less likely to have one disability condition than no disability, compared with those whose households used polluting cooking fuel. Also, compared with older adults whose households used polluting cooking fuel, older adults whose household used clean cooking fuel [aOR = 0.93; 95% CI = 0.90–0.96] were 7% less likely to have two or more disability conditions than no disability.

## Discussion

We examined the prevalence and factors associated with disability in older adults in Ghana. This is in light of the scant literature on the issue in Ghana. The results indicate that the prevalence of disability in older adults in Ghana is 38.4%. This implies that one-third of respondents had at least one disability condition and this calls for immediate attention such as improvement in health and social services to reduce the disability burden of older adults. In this study, the proportion of disability is higher than that of the general population (7.8%) according to the 2021 GPHC [4]. However, the prevalence of disability as found in this study is lower than those found in other studies. For instance, Biritwum et al.’s [18] multi-country study found that the prevalence of disability in Ghana was (85.4%), South Africa (77.0%), China (69.6%), India (93.2%), Russia (91.8%), and Mexico (81.5%). Also, Darkwah et al.’s [19] study in Ghana found that the prevalence of disability (functional limitation) was 44.6%. The disparities in the prevalence of disability could be attributed to differences in the instruments used to measure disability. Biritwum et al.’s [18] study used the 12-item World Health Organisation Disability Assessment Schedule (WHODAS II) to measure disability, while Darkwah et al.’s [19] study used the 9-item World Health Organisation Disability Assessment Schedule (WHODAS II) to measure disability. However, the present study used the Washington Group questions on disability to measure disability.

Our findings showed that female older adults had higher likelihood of disability than their male counterparts. Similar findings have been reported in South Africa [33], Malawi [34], a multi-country study [35] and other developing countries [22, 36]. However, Wandera et al.’s [15] study in Uganda found no relationship between gender and disability in older adults. The gender differentials in disability have been linked to socio-economic inequalities between men and women [22]. In that, the low socio-economic status of women could predispose them to disability conditions. Xavier Gómez-Olivé et al. [27] further explained that women may be more aware of their health status compared to men and thus may report health challenges in higher proportions than men. This could therefore explain a higher incidence of disability in female older adults than male older adults. In addition, higher life expectancy of women and increase incidence of non-fatal disabling conditions including depression and fractures have been noted to exert an influence on disability conditions on women [30]. This implies that there should be an effort to improve the socio-economic status of women by empowering them through education and other income-generating programmes to improve their economic status. Hypertension and arthritis should be priorities for action in Ghana since it is prevalent among older adults, especially females [37].

The findings also revealed that the level of disability increases as an individual advances in age. This is comparable to other studies in Ghana [19], Uganda [15], Nigeria [38], Tanzania [39] and a multi-country study [35]. The process of ageing is associated with physiological system decline including nervous and musculoskeletal systems which aid in carrying out daily activities. Also, there is a functional decline, sensory loss and increased likelihood of getting chronic non-communicable diseases [17]. This explains the finding that at older ages (70 years and above), individuals have a higher likelihood to experience disability than those aged 60 to 69 years. This calls for urgent policy interventions aimed at improving the health of older adults through early prevention and management of chronic non-communicable conditions in Ghana.

From the findings, having JHS/Middle, SHS and tertiary education was a protective factor against having two or more disability conditions. Education enhances individuals’ access and understanding of health information, which could reduce their likelihood of developing disabilities [40, 41].

Further, the findings showed that the likelihood of having disability was lower for married older adults than for those ever or never married. Similar findings have been reported elsewhere [15, 26, 27, 42]. In African tradition, particularly in Ghana, institutional care of older adults is rare, and therefore the majority of older adults are taken care of by their family members. In the past, older adults were catered for by the extended family, however, due to modernisation, urbanisation and education, there is a changing pattern of care, where older adults are cared for by their spouse or children. Hence, married older adults may receive emotional and physical support from their partners when they experience health conditions than unmarried women. Receiving emotional and physical support from partners could reduce loneliness and depression which has been reported to be associated with cognitive declines in older adults [43–46]. Thus, the absence of a partner could lead to isolation, lack of support and emotional depression and consequently lack of general health care for unmarried older adults which could account for disability conditions. Policy interventions should therefore target older adults who are ever or never married to help reduce their likelihood of being disabled.

It was also evident in our findings that rural residents have a higher likelihood of being disabled than urban residents. Living in rural areas has been associated with a higher likelihood of disability in older people in Uganda [15], Ghana [19], and a multi-country study [35]. This finding has been attributed to rural-urban differentials in socio-economic status including less education, poor living conditions and access to healthcare and preventive services, which is to the disadvantage of rural residents [19]. This suggests a need to reduce socio-economic inequalities in rural and urban areas in Ghana to help improve the wellbeing of older adults in rural areas. There is a need to establish more health facilities in rural areas to improve health care coverage and access to health care services. In addition, it is essential to ensure that health facilities in rural areas have sufficient and qualified health professionals to provide primary and specialised health care services to satisfy the needs of the sick, including older adults.

Older adults who resided in the Coastal zone were more likely to have a disability condition and two or more disability conditions than those who resided in the Middle and Northern zones This finding is unsurprising since most formal workers in Ghana are located in the Greater Accra region and Central region, which are part of the Coastal zone [47]. Formal work environment is associated with sedentary behaviour, such as prolong sitting and low physical activity, which can consequently lead to poor health, including the increase likelihood of disability [48–51].

Regarding employment status, unemployed older adults had a higher likelihood of being disabled than their counterparts who were employed. This finding is similar to studies in South Africa [27] and a multi-country study [35]. Employed older adults may be less likely to have disability conditions since they have more economic resources to access better healthcare services than those unemployed. Also, older adults with disabilities might have left their jobs due to their disability conditions. Studies have linked more economic resources with access to better healthcare services [52–54].

Older adults with health insurance were more likely to have disability conditions than those without health insurance. A plausible explanation is that older adults may have disabilities, which motivates them to enrol or renew their national health insurance so they can access adequate healthcare when needed.

Also, it was found that being affiliated with a religious group lowers one’s likelihood of being disabled. This finding is consistent with previous studies that have linked religion with better health [55–57]. A plausible explanation is that association with a religious group has a positive influence on healthy lifestyles education such as increased physical activity, healthy eating, non-use of alcohol and tobacco compared to those who do not associate with any religious group [58–60]. This is because religious groups occasionally organise physical activity and health talks on such issues at their place of worship [61, 62]. Besides, members of religious groups sometimes receive support from co-members to practice healthy behaviour [63].

Also, older adults from poor households had a lower likelihood of being disabled than those from rich households. This finding is in contrast with studies by Makaroun et al. [64] and Rahman et al. [35] who reported an inverse relationship between wealth and disability. In order to validate our findings, future quantitative studies should use longitudinal data to replicate our study since disability and household wealth could be bidirectional [65, 66]. Concurrently, qualitative studies could be conducted to delve into how household wealth might influence disability and vice versa. However, survivorship bias may explain why older adults from rich households had a higher likelihood of disability than those from poor households. That is to say, older adults from poor households with a disability may be less likely to live into older adulthood.

In addition, the use of clean cooking fuel was a protective factor against having a disability condition. This finding corroborates previous studies which found association between polluting cooking fuel (such as biomass), functional disabilities and cognitive impairments [67–69]. The use of polluting cooking fuel is associated with adverse health outcomes, such as respiratory infections, skin diseases, ophthalmic symptoms, and cardiovascular diseases which can increase one’s likelihood of having a disability condition [70, 71]. The adverse consequences associated with using polluting cooking fuel necessitate implementing initiatives to advocate for the adoption of clean cooking fuel in households across the entire nation.

## Strength and limitations of the study

The main strength of this study is its large sample size. The study used a national census, and therefore, the findings of the study can be generalised. Also, this study measured six domains (physical, sight, intellectual, hearing, self-care, and speech) of disability in older adults. This study also contributes to the scanty literature on the prevalence and factors associated with disability in older adults in Ghana. However, there are limitations to this study. One limitation is that the presence of chronic non-communicable disease is a key factor that might have an influence on disability in older adults. However, we could not examine its effect on disability due to data limitation. Also, the data uses a cross-section design and thus we cannot draw conclusions of causality between the predictor variables and disability status. Despite these limitations, the findings contribute to filling the knowledge gap on the issue of disability in older persons in Ghana.

## Conclusion

This study found that 38.36% of the older adults in Ghana were disabled. Also, socio-demographic (such as age, region of residence, place of residence, health insurance status, and marital status, among others) and household (such as cooking fuel, and household wealth) characteristics were correlates of disability in the respondents. So, policymakers and researchers should target these factors when designing appropriate policies, programmes, and interventions to improve the wellbeing of older adults.

### Electronic supplementary material

Below is the link to the electronic supplementary material.

Supplementary Material 1

## Data Availability

The datasets generated and/or analysed during the current study are available at the Ghana Statistical Service database at repository: https://www2.statsghana.gov.gh/nada/index.php/catalog/110.

## Abbreviations

aOR: Adjusted Odds Ratio
CAPI: Computer Assisted Personal Interview
CI: Confidence Interval
EA: Enumeration Areas
GPHC: Ghana Population and Housing Census
JHS: Junior High School
RC: Reference Category
SHS: Senior High School
WHODAS: World Health Organisation Disability Assessment Schedule
