# Supplementary material for: Prevalence and correlates of disability in older adults, Ghana: evidence from the Ghana 2021 Population and Housing Census
Source: BMC Geriatr. 2024 Jan 11;24:52. doi: 10.1186/s12877-023-04587-6 (PMC10785330; doi:10.1186/s12877-023-04587-6)
Supplement: Supplementary file 1 — Supplementary Material 1 [file 12877_2023_4587_MOESM1_ESM.docx]

**Table S1: Definition of variables**

| **Variables** | **Categories** | **Definitions** |
| --- | --- | --- |
| **Age** | 1. 60-69 | Age of respondents was measured by the ages of older adults during the census night. This was categorised into 60-69 years, 50-79 years, and 80 years and above. |
|  | 1. 70-79 |  |
|  | 1. 80 and above |  |
| **Sex** | 1. Male | Sex of respondents was measured by either being male or female. |
|  | 1. Female |  |
| **Education** | 1. No education | Education was measured by the highest educational level attained by the older adults. |
|  | 1. Primary |  |
|  | 1. JHS/Middle |  |
|  | 1. SHS |  |
|  | 1. Tertiary |  |
| **Marital status** | 1. Currently married | Marital status of respondents was measured by the current marital status of older adults. Ever married category consist of older adults who are widowed, had separated or divorced. |
|  | 1. Ever married |  |
|  | 1. Never married |  |
| **Employment status** | 1. Employed | Employment status was measured an engagement in any economic activity seven (7) days before the census night (21^st^ – 27^th^ June, 2021) for at least an hour. Older adults who responded yes were classified as employed, and those who responded no were classified as unemployed. |
|  | 1. Unemployed |  |
| **Region of residence** | 1. Southern | Region of residence was measured by the region where older adults lived at the census night. Greater Accra, Oti, Volta, Central, Western, Western North regions were reclassified as Southern zone. Eastern, Ashanti, Bono East, Ahafo, and Bono regions were reclassified as Middle zone, while Savanna, Northern, Upper East, Upper West and North East regions were reclassified as Northern zone. |
|  | 1. Middle |  |
|  | 1. Northern |  |
| **Place of residence** | 1. Urban | Place of residence was measured by place within a region where the older adults resided at the census night. Ghana Statistical Service defined a rural area as an area with less than 5,000 population. |
|  | 1. Rural |  |
| **Religious affiliation** | 1. Affiliated | Religious affiliation was measured by the religious group older adults belonged to. Those who belonged to Christianity, Islam, Traditional beliefs and practices, and other religious groups were reclassified as religiously affiliated. On the other hand, those who did not belong any religious group were reclassified as religiously unaffiliated. |
|  | 1. Unaffiliated |  |
| **Household wealth** | 1. Poor | Household wealth of respondents was measured by other adults’ household ownership of assets. These assets include radio/stereo, television (digital and analog), telephone (fixed and cordless), bicycle, canoe, outboard motor, fridge, deep freezer, desktop computer, laptop, tricycle, motor cycle, private car/truck, tractor, cart, donkey/camel/mule, and home theatre. The principal components analysis was used to generate factor scores for each household asset and a standardised score was used to divide the household wealth score into five categories: lowest, second, middle, fourth, and highest. The lowest and second were reclassified as poor, while the fourth and highest were reclassified as rich. The middle was left. Consequently, the household wealth had three categories: poor, middle and rich. |
|  | 1. Middle |  |
|  | 1. Rich |  |
| **Health insurance status** | 1. Yes | Health insurance status was measured by whether or not the older adults paid or renewed their annual premium of national health insurance scheme or a private insurance. Those who have paid or renewed their annual insurance premium were categorised as yes, while those who had not enrolled unto an insurance scheme or not paid or renewed their annual insurance premium were categorised as no. |
|  | 1. No |  |
| **Cooking fuel** | 1. Polluting | Cooking fuel was measured by the main source of cooking fuel for older adults’ households. Liquified petroleum gas, biogas, electricity and cooking gel were reclassified as clean cooking fuel, while wood, kerosene, charcoal, crop residue, sawdust and animal waste were reclassified as polluting cooking fuel. |
|  | 1. Clean |  |
